# Impact of 17β-Estradiol on the Shape, Survival, Osteogenic Transformation, and mRNA Expression of Gingiva-Derived Stem Cell Spheroids †

**DOI:** 10.3390/medicina60010060

**Published:** 2023-12-28

**Authors:** Ju-Hwan Kim, Hyun-Jin Lee, Hye-Jung Song, Jun-Beom Park

**Affiliations:** 1 Department of Periodontics, College of Medicine, The Catholic University of Korea, Seoul 06591, Republic of Korea; juhwank33@naver.com (J.-H.K.); hyunjinlee0423@gmail.com (H.-J.L.); 2 Graduate School of Clinical Dental Science, The Catholic University of Korea, Seoul 06591, Republic of Korea; hjsong55@catholic.ac.kr; 3 Dental Implantology, Graduate School of Clinical Dental Science, The Catholic University of Korea, Seoul 06591, Republic of Korea; 4 Department of Medicine, Graduate School, The Catholic University of Korea, Seoul 06591, Republic of Korea

**Keywords:** cell differentiation, cell survival, estradiol, osteogenesis, stem cells

## Abstract

*Background and Objectives*: Mesenchymal stem cells hold promise for tissue regeneration, given their robust growth and versatile differentiation capabilities. An analysis of bone marrow-sourced mesenchymal stem cell proliferation showed that 17β-estradiol could enhance their growth. This study aims to investigate the influence of 17β-estradiol on the shape, survival, osteogenic differentiation, and mineralization of human mesenchymal stem cells. *Materials and Methods*: Spheroids made from human gingiva-derived stem cells were cultivated with varying concentrations of 17β-estradiol: 0, 0.01, 0.1, 1, and 10 nM. Morphology was assessed on days 1, 3, and 5. The live/dead kit assay was employed on day 3 for qualitative cell viability, while cell counting kit-8 was used for quantitative viability assessments on days 1, 3, and 5. To evaluate the osteogenic differentiation of the spheroids, a real-time polymerase chain reaction assessed the expressions of RUNX2 and COL1A1 on day 7. *Results*: The stem cells formed cohesive spheroids, and the inclusion of 17β-estradiol did not noticeably alter their shape. The spheroid diameter remained consistent across concentrations of 0, 0.01, 0.1, 1, and 10 nM of 17β-estradiol. However, cellular viability was boosted with the addition of 1 and 10 nM of 17β-estradiol. The highest expression levels for RUNX2 and COL1A1 were observed with the introduction of 17β-estradiol at 0.1 nM. *Conclusions*: In conclusion, from the results obtained, it can be inferred that 17β-estradiol can be utilized for differentiating stem cell spheroids. Furthermore, the localized and controlled use, potentially through localized delivery systems or biomaterials, can be an area of active research. While 17β-estradiol holds promise for enhancing stem cell applications, any clinical use requires a thorough understanding of its mechanisms, careful control of its dosage and delivery, and extensive testing to ensure safety and efficacy.

## 1. Introduction

Recently, there has been a significant increase in interest and funding for stem cell research and its potential medical uses [1,2,3]. Stem cells are, incredibly, capable of differentiating and self-renewing [4]. Furthermore, stem cells are at the forefront of biomedical innovation due to their exceptional capacities for producing regenerative medicine and to differentiate into a variety of cell types [5]. In particular, stem cells present a promising avenue for replacing damaged tissues and organs in medical research, potentially circumventing the constraints of existing treatments and providing cures for patients suffering from acute injuries and chronic illnesses [6]. Stem cells provide a platform for disease modeling and drug screening beyond direct therapy, which can result in more convenient methods with less adverse effects [7,8]. By encouraging patient pluripotent stem cells to develop into cell types impacted by a specific disease, scientists can create disease-in-a-dish or lab-on-a-chip models that help them understand the mechanisms behind the condition and identify possible treatment targets [9,10]. Because of these cells’ adaptability, research was conducted to better understand their biology, manipulate them in the laboratory, and ensure that they are used safely and effectively in clinical settings [11].

Gingival-derived stem cells are gaining attention for their potential use in regenerative medicine and tissue engineering due to several key properties [12]. Gingival-derived stem cells are a type of stem cell derived from gingival tissue, the gum tissue that surrounds the teeth [13]. Gingival-derived stem cells are easily accessible and can be obtained through minimally invasive procedures, making them an attractive source of stem cells compared to other types of stem cells that must be extracted through more invasive methods, such as bone marrow stem cells [14]. Gingival-derived stem cells are reported to present a high rate of cell division and proliferation and can generate significant numbers of cells within a short period of time [15]. Gingival-derived stem cells are pluripotent, meaning they have the ability to differentiate into a variety of cell types, including osteoblasts [16]. Gingival-derived stem cells have an immunomodulatory capacity, which means they can modulate the immune response, providing the benefits of reducing inflammation and preventing rejection in tissue-engineered constructs and grafts [17]. Due to these properties, gingival-derived stem cells can be considered promising for regenerative medicine applications, such as periodontal tissue regeneration, bone regeneration, and potentially wound healing and other damaged tissue repair [18].

A kind of estrogen called 17β-estradiol is known to affect several types of cells, including stem cells [19]. Estrogen can help stem cells survive under challenging circumstances, like oxidative stress, when it comes to stem cell treatment applications [20]. It has been demonstrated that estrogen promotes the growth of mesenchymal stem cells, which are essential for tissue regeneration [21]. Stem cells and other agents, such as growth factors, may work better together to promote the functionality aspect [22,23]. Similarly, previous research demonstrated that mesenchymal stem cells benefited with the addition of growth factors [24]. Three-dimensional culture systems are emerging as an important tool for studying the behavior and applications of stem cells [25]. Unlike traditional two-dimensional monolayer cultures, three-dimensional culture platforms provide a more physiologically relevant microenvironment, which can better mimic in vivo conditions [26]. Moreover, spheroid culture, a form of three-dimensional cell culture, has also emerged as a useful model for studying the behavior and interactions of cells in microenvironments that mimic in vivo conditions [27]. Spheroid culture allows cells to self-organize into three-dimensional structures with unique properties [28]. The cells in a spheroid exhibit a gradient of cell density with a proliferative outer layer and a dormant core, and spheroid culture mimics the natural nutrient and oxygen gradients of tissues [29]. Stem cells can be employed in numerous research situations alongside 17β-estradiol [20]. 17β-estradiol can encourage stem cells to differentiate into a variety of lineages, such as osteoblasts, which are crucial for bone homeostasis, repair, and regeneration, according to some data [30]. This study aims to investigate the influence of 17β-estradiol on the shape, survival, osteogenic differentiation, and mineralization of human mesenchymal stem cells.

## 2. Materials and Methods

### 2.1. Design of the Present Study with Gingiva-Derived Mesenchymal Stem Cells and Fabrication of Stem Cell Spheroids

The Institutional Review Board of Seoul St. Mary’s Hospital, College of Medicine, The Catholic University of Korea, examined and approved this research protocol (KC22SISE0170 approved on 15 March 2022 and KC23SISE0398 approved on 13 October 2023). The participant provided the consent. Every experiment was carried out in compliance with the applicable rules and specifications listed in the Declaration of Helsinki.

Gingival tissues were initially preserved in a sterile solution of phosphate-buffered saline supplemented with penicillin (100 U/mL) and streptomycin (100 µg/mL) from Sigma-Aldrich Co., St. Louis, MO, USA, and maintained at a temperature of 4 °C [31]. These tissues underwent the removal of the epithelial layer, were finely chopped into pieces measuring 1–2 mm^2^, and then treated with a mixture of 0.2 µm of filtered alpha-modified minimal essential medium (a-MEM; produced by Gibco, Grand Island, NY, USA), dispase (1 mg/mL), and collagenase IV (2 mg/mL), both obtained from Sigma-Aldrich Co. This digestion process occurred at 37 °C for 30 min. The first batch of cells released during this process was discarded, and the tissues were subjected to an additional 90 min digestion period under the same conditions. The resulting cell mixture was then passed through a 70 μm cell strainer (brand Falcon, Franklin Lakes, NJ, USA) and cultured in α-MEM enriched with 15% fetal bovine serum (from Gibco), penicillin (100 U/mL), streptomycin (100 µg/mL), L-glutamine (200 mM), and ascorbic acid 2-phosphate (10 mM), all supplied by Sigma-Aldrich Co. This cell culture was housed in a 75 cm^2^ tissue culture flask produced by Corning, Tewksbury MA, USA, and incubated at 37 °C in a humidity-controlled environment with a mix of 5% CO_2_ and 95% air. After 24 h, non-adhering cells were removed using phosphate-buffered saline; the medium was refreshed. The culture was nourished every two to three days and the cells were grown in an incubator.

Stem cells obtained from gingiva were cultivated in an osteogenic medium after being plated onto 600 µm diameter concave microwells (StemFIT 3D; MicroFIT, Seongnam-si, Gyeonggi-do, Republic of Korea) composed of silicon elastomer at a density of 1 × 10^6^ cells/well [32]. The final concentrations of 17β-estradiol (3301-1GM, Sigma-Aldrich, St. Louis, MO, USA) were 0, 0.01, 0.1, 1, and 10 nM. Using an inverted microscope, the morphological examination was completed on days 1, 3, and 5 (CKX41SF, Olympus Corporation, Tokyo, Japan). 

### 2.2. Determination of Qualitative and Quantitative Cell Viability

Day 3 of the cultivation of cell spheroids in osteogenic media was used to assess the qualitative cell viability using the live/dead kit assay (Molecular Probes, Eugene, OR, USA) [32]. This assay distinguishes between live and dead cells based on the integrity of the cell membrane and overall cellular health. It typically uses a fluorescent dye that can selectively stain live and dead cells, producing a distinct fluorescence pattern that can be visualized and quantified by fluorescence microscopy or flow cytometry [33]. Calcein AM dye is taken up by living cells with intact cell membranes. Intracellular esterases cleave calcein AM to produce a green fluorescent compound once inside the cell. Ethidium homodimer-1 is a fluorescent dye commonly used in live/dead cell viability assays to stain and identify dead cells within a cell population. Ethidium homodimer-1 plays a specific role in these assays by selectively staining cells with a compromised membrane integrity, a characteristic of dead or dying cells. A fluorescence microscope was used to view these spheroids at a ×100 magnification after they had been cultured for 60 min at room temperature (Axiovert 200, Carl Zeiss, Oberkochen, Germany). Using cell counting kit-8 (Dojindo, Tokyo, Japan), a quantitative cell viability test was conducted on days 1, 3, and 5 [34]. Cell counting kit-8 is an assay kit commonly used in cell biology and life sciences to assess cell viability, proliferation, and cytotoxicity. It is a colorimetric assay that quantitatively measures the number of viable cells in a cell population based on the metabolic activity of the cells. The cell counting kit-8 assay relies on the fact that viable cells have a metabolic activity, specifically in the mitochondria. In living cells, mitochondrial enzymes reduce the water-soluble tetrazolium salt in the cell counting kit-8 reagent to the formazan dye, resulting in a color change [35]. Cell counting kit-8 is widely used in research, drug development, and toxicity testing.

### 2.3. Total RNA Extraction and Quantification of RUNX2 and COL1A1 mRNA by Real-Time Quantitative Polymerase Chain Reaction (qPCR)

On the seventh day, mRNA expression levels were determined using qPCR. Total RNA was isolated using a kit provided by Thermo Fisher Scientific, Inc., Waltham, MA, USA, following the guidelines supplied by the manufacturer [36]. The RNA concentration was measured using a spectrophotometer (ND-2000, Thermo Fisher Scientific, Inc.) and a bioanalyzer (Agilent 2100), employing the RNA 6000 Nano Chip kit from Agilent Technologies, to determine the absorbance at wavelengths of 260 and 280 nm. Reverse transcription was performed using RNA as the template with the reverse transcriptase enzyme SuperScript II from Invitrogen, Carlsbad, CA, USA.

For the PCR process, sense and antisense primers were designed based on sequences from GenBank. The primer sequences for RUNX2 (accession No.: NM_001015051.3) were forward primer 5′-CAGTTCCCAAGCATTTCATCC-3′ and reverse primer 5′-AGGTGGCTGGATAGTGCATT-3′. The primer sequences for COL1A1 (accession No.: NM_000088.4) were forward primer 5′-TACCCCACTCAGCCCAGTGT-3′ and reverse primer 5′-CCGAACCAGACATGCCTCTT-3′. For β-actin (accession No.: NM_001101), the primer sequences were forward primer 5′-AATGCTTCTAGGCGGACTATGA-3′ and reverse primer 5′-TTTCTGCGCAAGTTAGGTTTT-3′ [37,38].

### 2.4. Statistical Analysis

The data are reported as the mean ± standard deviation. To assess the distribution and variance homogeneity, normality and equality of variance tests were applied. Group comparisons were executed using the one-way analysis of variance (ANOVA), followed by Tukey’s post hoc test for multiple comparisons. Each analysis was replicated three times experimentally. 

## 3. Results

### 3.1. Cell Spheroids of Human Gingiva-Derived Mesenchymal Stem Cells

**Figure 1:**
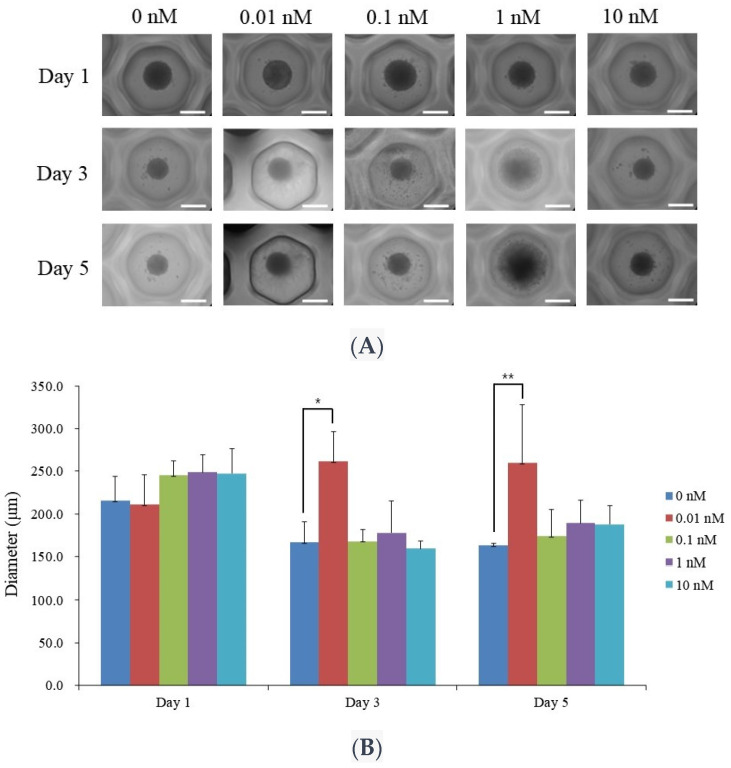
Morphological evaluation. (**A**) The morphologies of stem cell spheroids treated with 17β-estradiol at 0, 0.01, 0.1, 1, and 10 nM concentrations on days 1, 3, and 5. The scale bar represents 200 μm (original magnification ×200). (**B**) The diameters of the stem cell spheroids on days 1, 3, and 5. * *p* < 0.05 on day 3 compared to the time-matched unloaded group. ** *p* < 0.05 on day 5 compared to the time-matched control group.

Figure 1A depicts the morphologies of stem cell spheroids treated with 17β-estradiol of final concentrations of 0, 0.01, 0.1, 1, and 10 nM on days 1, 3, and 5. The spheroid appeared compact with a clear boundary. The spheroid maintained its compact structure similar to the control, irrespective of the increase in the 17β-estradiol concentration. Similarly, there was no apparent change in the spheroid’s central region along the different concentration of 17β-estradiol. The structure along with the center of stem cell spheroids did not show noticeable changes over five days.

The changes in the cell diameter over a five-day period at varying concentrations of 17β-estradiol are shown in Figure 1B. There were no statistically significant differences between the groups. However, there was increase in the size of the spheroid in the 0.01 nM group on day 5. 

### 3.2. Qualitative Determination and Quantitative Values of Cellular Viability

**Figure 2:**
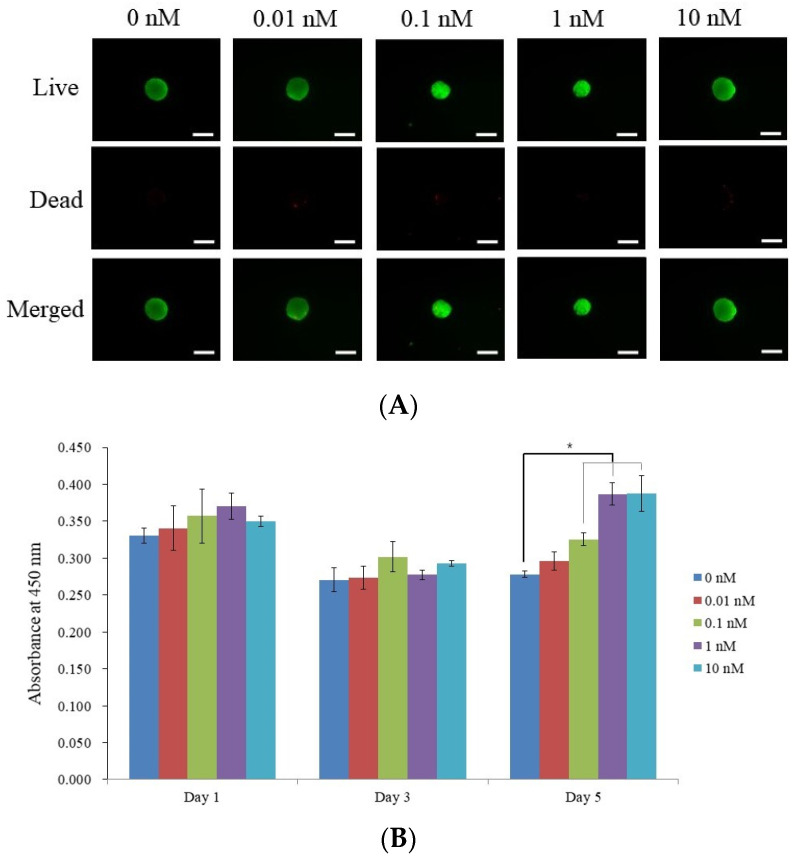
Cellular viability. (**A**) Images depicting optical, live, dead, and combined views of stem cell spheroids on the third day. A scale bar indicating 100 μm is included (with an original magnification of ×100). (**B**) Assessment of cell viability utilizing cell counting kit-8 on days 1, 3, and 5. * *p* < 0.05 on day 5 compared to the time-matched unloaded control group.

On the third day, the viability of stem cells was assessed qualitatively using a live/dead kit assay. Figure 2A showcases a series of fluorescence microscope images that provide insights into the viability of stem cell spheroids when treated with different concentrations of 17β-estradiol. The figure provides color indicators to differentiate between live (green) and dead cells (red), with the merged images offering a combined view. The result in the 0 nM group shows a well-defined green spheroid, which indicates a significant number of living cells along with minimal to no red fluorescence, suggesting few dead cells. The spheroid’s green fluorescence is consistent at 0.1 nM along with the other concentrations. The merged images show the image is mainly green with minimal visible red regions.

All concentrations, including the control, exhibited similar absorbance values, suggesting comparable cell viability across the groups on the first day (Figure 2B). There was a slight decrease in the absorbance values for all groups compared to day 1. This suggests a decrease in the cell viability over time, although the differences between the concentrations remain relatively minimal. A notable observation was the higher absorbance value, which was indicative of increased cell viability values for the 0.1, 1, and 10 nM concentrations. The highest value was obtained for the 10 nM concentration with statistical significance (*p* < 0.05).

### 3.3. Evaluations of RUNX2 and COL1A1 by qPCR

**Figure 3:**
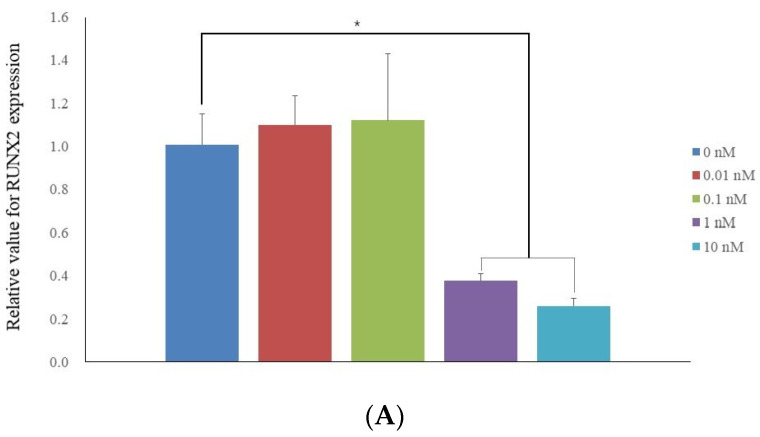

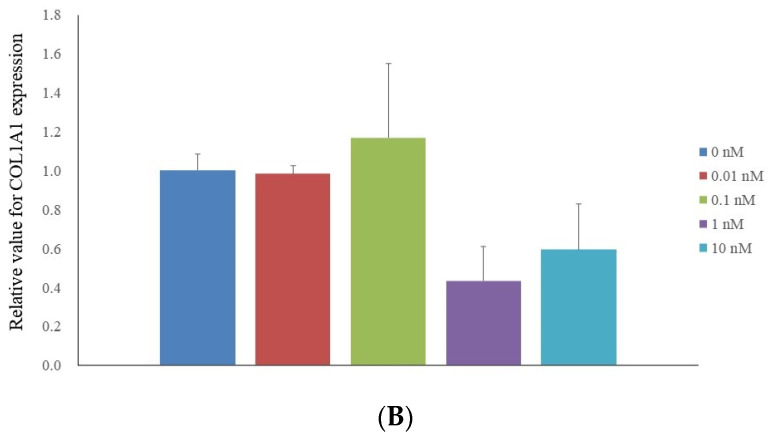
mRNA expression. (**A**) Quantification of expression of RUNX2 mRNA by real-time polymerase chain reaction on day 7. (**B**) Quantification of expression of COL1A1 mRNA by real-time polymerase chain reaction on day 7. * *p* < 0.05 on day 7 compared to the time-matched unloaded group.

qPCR revealed that the mRNA levels of RUNX2 on day 7 were 1.007 ± 0.144, 1.099 ± 0.133, 1.119 ± 0.310, 0.377 ± 0.033, and 0.259 ± 0.035 for 17β-estradiol at 0, 0.01, 0.1, 1, and 10 nM, respectively (*p* < 0.05) (Figure 3A). The highest value was obtained for the 0.1 nM group.

The addition of 17β-estradiol led to the expressions of COL1A1 as 1.002 ± 0.084, 0.983 ± 0.042, 1.170 ± 0.380, 0.435 ± 0.177, and 0.595 ± 0.233 for 17β-estradiol at 0, 0.01, 0.1, 1, and 10 nM, respectively (*p* < 0.05) (Figure 3B). The highest expression was obtained for the 0.1 nM group.

## 4. Discussion

This research analyzed the effects of 17β-estradiol on the osteogenic differentiation and mineralization of human mesenchymal stem cells. Cellular viability was assessed using cell counting kit-8, and differentiation into an osteogenic lineage was performed. The mRNA levels of RUNX2 and COL1A1 were detected using a real-time quantitative polymerase chain reaction. Our study explored the effects of 17β-estradiol on human gingiva-derived mesenchymal stem cell spheroids. The consistent spheroid shape across all 17β-estradiol concentrations suggested that the hormone did not induce morphological changes under the conditions tested. A notable increase in the cellular viability at higher concentrations of 17β-estradiol (1 and 10 nM) aligned with the studies demonstrating estrogen’s role in enhancing cell survival and proliferation [39]. Estrogen has been reported to promote the survival of osteoblasts, preventing their apoptosis, and this is vital for ensuring a sufficient number of osteoblasts for bone formation and repair [40].

Furthermore, 17β-estradiol has been shown to have both direct and indirect effects on bone cells, influencing osteoblasts and osteocytes [41,42]. Additionally, 17β-estradiol has been shown to stimulate the differentiation of mesenchymal stem cells into osteoblasts, bone-forming cells [43]. The observed upregulation of osteogenic markers RUNX2 and COL1A1, particularly at 0.1 nM, could indicate a hormonally facilitated enhancement in osteogenic potential [44]. The exposure to 17β-estradiol enhanced the deposition of the bone matrix by osteoblasts and, more specifically, 17β-estradiol treatment was linked to the increased synthesis of collagen, the primary structural protein in the bone matrix [45]. 

Selecting an optimal dosage is very important. The dosages of 17β-estradiol were applied at 0.0001 and 0.01 nM [46]. A previous study used a physiological dose of 0.1 nM and infraphysiological dose of 1 nM [41]. In another study, the dosages of 17β-estradiol used were 10, 50, 100, 500, 1, 10, and 50 mM [47]. The least effective concentration of 17β-estradiol that could promote potent anti-inflammatory properties in the mesenchymal stem cell population was 100 nM [48]. Lower concentrations (0.01 and 1 nM) appeared to promote RUNX2 expression, but higher quantities of the drug (1 and 10 nM) decreased the expression. In this study, the cell viability increased in a dose-dependent manner, with the greatest effects at 10 nM. Otherwise, higher quantities of 17β-estradiol (1 and 10 nM) significantly inhibited RUNX2 mRNA expression, but lower concentrations (0.01 and 0.1 nM) appeared to promote it. Concentrations of 0.1 nM resulted in an increased expression of COL1A1 mRNA, whereas 1 and 10 nM showed decreased expressions. This implies that the compound influences RUNX2 and COL1A1 expressions in a biphasic manner, enhancing it at low concentrations and blocking it at high concentrations.

The action of 17β-estradiol can be explained by the following reasons. The transcription factor RUNX2 is necessary for the differentiation of osteoblasts and the formation of the skeleton [49]. The primary component of type I collagen, encoded by COL1A1, is essential for maintaining the structural integrity of many tissues, including skin and bone [50]. Moreover, 17β-estradiol is reported to play an anti-osteoporosis role via a novel ESR1-Keap1-Nrf2 axis-mediated regulation [51]. The Nrf2 knockout largely blocks the bone anabolic effect of 17β-estradiol in vivo and ex vivo, and this suggests estrogen prevents osteoporosis through promoting osteoblastic bone formation via the Nrf2-mediated activation of antioxidant signaling [51]. Furthermore, 17β-estradiol upregulates the PI3K-Akt signaling pathway to promote bone marrow mesenchymal stem cell angiogenic differentiation [52]. The osteogenic effects of 17β-estradiol are mediated through estrogen receptors, especially estrogen receptor-α, and the activation of these receptors can modulate various intracellular pathways, including PI3K/Akt and MAPK, which are crucial for osteoblast differentiation and function [53].

Combination therapy was applied to enhance functionality. The applications of 17β-estradiol and testosterone led to increased alkaline phosphatase activity, calcium composition, and osteogenic-related gene and protein expressions [47]. Additionally, 17β-estradiol can upregulate the expression of growth factors, such as the insulin-like growth factor-1, which further promotes osteoblast differentiation and bone formation [54]. The effectiveness of oral hormone therapy using 17β-estradiol alone, and in combination with norethindrone acetate, for preventing bone loss in women who have recently entered menopause was evaluated [55]. The previous study found that 17β-estradiol had a dose-related impact on improving bone mineral density. Furthermore, it appeared that the inclusion of norethindrone acetate amplified the improvement of bone mineral density seen with 17β-estradiol. Combination therapy with 17β-estradiol has been performed in other areas. Combination treatment with 17β-estradiol and therapeutic hypothermia for transient global cerebral ischemia in rats was performed, and the postischemic administration of low-dose 17β-estradiol appeared to be neuroprotective [56]. Combination therapy using 17β-estradiol and the recombinant tissue plasminogen activator was effective for experimental ischemic strokes [57].

There were some limitations to this study. The study only tested four concentrations, and additional concentrations, especially higher than 10 nM or lower than 0.01 nM, could provide further insights into the dose–response relationship. The concentration-dependent effects presented a non-linear relationship between 17β-estradiol and mesenchymal stem cell function. However, considering that we did not explore concentrations higher than 10 nM, we could not exclude the possibility of different effects at higher doses. Previous research indicates the biphasic responses of stem cells to hormonal treatment, necessitating a broader concentration range for a comprehensive dose–response analysis [58]. Observations were performed up to day 7, and it was possible that this time frame did not capture the longer-term impacts on stem cell spheroids, such as cellular survival and osteogenic differentiation. Moreover, our study’s brief length made it impossible to comprehend the long-term implications, such as the possibility of terminal differentiation and actual bone creation [59]. The study used a particular type of stem cell, and the effects of 17β-estradiol observed may not be generalizable to other stem cell types or to cells in a different physiological context [58]. The study assessed the mRNA expression levels of COL1A1 and RUNX2, which may not be a reliable indicator of the actual amounts or activity of the proteins [60]. We used an in vitro experimental approach, which restricted the direct applicability of our findings to in vivo settings where stem cell behavior was heavily influenced by the local microenvironment and systemic variables [61]. The study’s particular conditions could have limited the findings’ applicability to other experimental contexts, such as in vivo or clinical settings [62].

Despite these expectations, there are challenges when using stem cells [63]. The discussion and research continues on the technical challenges of controlling the differentiation of stem cells and controlling the expansion of these cells [64]. Regarding the broader implications of this study, our results suggest that 17β-estradiol contributes to the viability and differentiation of mesenchymal stem cells. Nevertheless, given the complex relationship of 17β-estradiol in the human body and the potential for systemic effects beyond the target cells, a thorough in vivo validation is required before these results can be put to practical use.

## 5. Conclusions

In conclusion, the results obtained allow us to infer that 17β-estradiol can be utilized for stem cell spheroid differentiation. Furthermore, its localized and controlled use via local delivery systems or biomaterials can potentially be an area of active re-exploration. Although 17β-estradiol has the potential to enhance stem cell applications, its clinical use requires a thorough understanding of its mechanisms, the careful control of dose and delivery, and extensive testing to ensure its safety and efficacy.

## Data Availability

This article contains all of the information that was created or examined during this investigation.
